# Supplementary material for: Linear RAG scanning mediates editing of Igκ variable region repertoires
Source: Nature. 2026 Apr 15;653(8115):870–8. doi: 10.1038/s41586-026-10362-5 (PMC13190342; doi:10.1038/s41586-026-10362-5)
Supplement: Supplementary file 1 — Gating strategy to determine BM B cell populations isolated from parental and edited chimeras. [file 41586_2026_10362_MOESM1_ESM.pdf]

nature portfolio

https://doi.org/10.1038/s41586-026-10362-5

Supplementary information

Linear RAG scanning mediates editing of Igκ variable region repertoires

In the format provided by the authors and unedited

Supplementary Figure

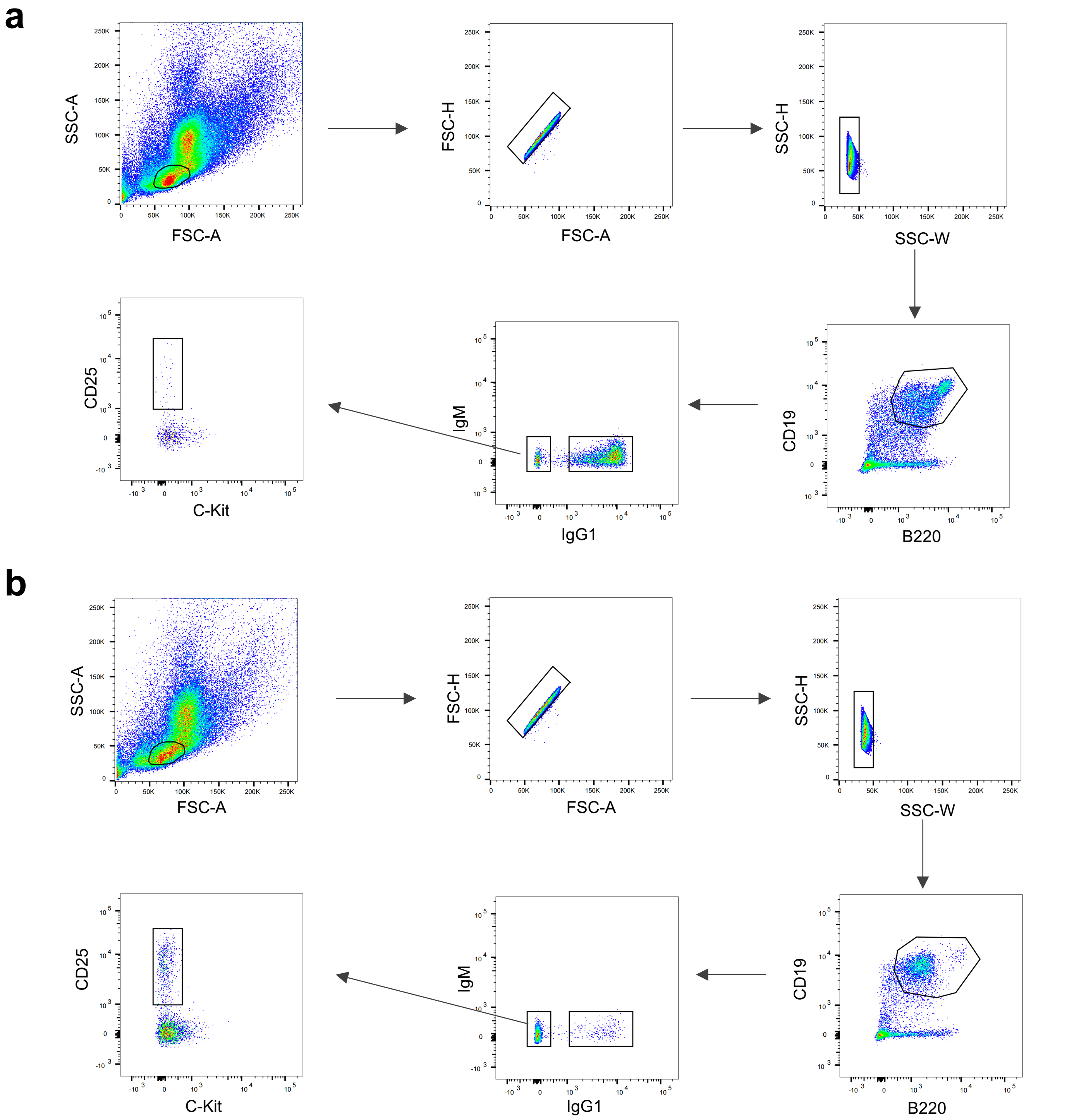

Supplementary Figure 1. Gating strategy to determine BM B cell populations isolated from parental (**a**) and edited (**b**) chimeras, as shown in the Extended Data Figure 5c and d respectively. $B220^{+}CD19^{+}IgM^{-}IgG1^{+}$ population and $B220^{+}CD19^{+}IgM^{-}IgG1^{-}CD25^{+}$c-Kit^{-} population were used for defining BM immature B cells and pre-B cells respectively.
