# Supplementary material for: Linear RAG scanning mediates editing of Igκ variable region repertoires
Source: Nature. 2026 Apr 15;653(8115):870–8. doi: 10.1038/s41586-026-10362-5 (PMC13190342; doi:10.1038/s41586-026-10362-5)
Supplement: Supplementary file 2 — Reporting Summary [file 41586_2026_10362_MOESM2_ESM.pdf]

nature portfolio

Corresponding author(s): Frederick W. Alt
Last updated by author(s): Feb 21, 2026

Reporting Summary

Nature Portfolio wishes to improve the reproducibility of the work that we publish. This form provides structure for consistency and transparency in reporting. For further information on Nature Portfolio policies, see our Editorial Policies and the Editorial Policy Checklist.

Statistics

For all statistical analyses, confirm that the following items are present in the figure legend, table legend, main text, or Methods section.

n/a	Confirmed	
☐	☒	The exact sample size (*n*) for each experimental group/condition, given as a discrete number and unit of measurement
☐	☒	A statement on whether measurements were taken from distinct samples or whether the same sample was measured repeatedly
☐	☒	The statistical test(s) used AND whether they are one- or two-sided<br>*Only common tests should be described solely by name; describe more complex techniques in the Methods section.*
☒	☐	A description of all covariates tested
☐	☒	A description of any assumptions or corrections, such as tests of normality and adjustment for multiple comparisons
☐	☒	A full description of the statistical parameters including central tendency (e.g. means) or other basic estimates (e.g. regression coefficient) AND variation (e.g. standard deviation) or associated estimates of uncertainty (e.g. confidence intervals)
☐	☒	For null hypothesis testing, the test statistic (e.g. *F*, *t*, *r*) with confidence intervals, effect sizes, degrees of freedom and *P* value noted<br>*Give P values as exact values whenever suitable.*
☒	☐	For Bayesian analysis, information on the choice of priors and Markov chain Monte Carlo settings
☒	☐	For hierarchical and complex designs, identification of the appropriate level for tests and full reporting of outcomes
☒	☐	Estimates of effect sizes (e.g. Cohen's *d*, Pearson's *r*), indicating how they were calculated

Our web collection on statistics for biologists contains articles on many of the points above.

Software and code

Policy information about availability of computer code

Data collection	Next generation sequencing data were collected via Illumina sequencing platforms (NextSeq 550 and NextSeq 2000). NextSeq 550 control software (2.2.0) and NextSeq 1000/2000 control software (1.5.0.42699) were used for high-throughput sequencing data collection. Data generated from NextSeq 550 or NextSeq 2000 were demultiplexed via TranslocPreprocess.pl, a published pipeline available at http://robinmeyers.github.io/transloc_pipeline/.
Data analysis	HTGTS-V(D)J-seq and 3C-HTGTS data were processed via the published pipeline (http://robinmeyers.github.io/transloc_pipeline/). The pipeline for analyzing 3C-HTGTS data is available at https://github.com/Yyx2626/HTGTS_related. The pipeline for GRO-seq data analysis is available at https://github.com/Yyx2626/Fred_Alt_Lab/tree/master/GROseq. The pipeline for ATAC-seq data analysis is available at https://github.com/nf-core/atacseq. The mouse Igk-specific cryptic RSS usage analysis pipeline is available at https://github.com/Yyx2626/HTGTS_related/tree/main/Igk_specific_anno_and_filter. GraphPad Prism 9 and R 3.6.1 were used for statistical analysis and graph visualization. IGV (2.11.1) was used to visualize RAG off-target data. FlowJo (version9.3.2) was used for analyzing the FACS data.

For manuscripts utilizing custom algorithms or software that are central to the research but not yet described in published literature, software must be made available to editors and reviewers. We strongly encourage code deposition in a community repository (e.g. GitHub). See the Nature Portfolio guidelines for submitting code & software for further information.

Data

Policy information about availability of data

All manuscripts must include a data availability statement. This statement should provide the following information, where applicable:

- Accession codes, unique identifiers, or web links for publicly available datasets
- A description of any restrictions on data availability
- For clinical datasets or third party data, please ensure that the statement adheres to our policy

HTGTS-V(D)J-Seq, 3C-HTGTS and GRO-seq sequencing data reported in this study have been deposited in the ArrayExpress database under the accession number E-MTAB-16001 for HTGTS-V(D)J-Seq data, E-MTAB-16007 for 3C-HTGTS data and E-MTAB-16014 for GRO-seq data. ATAC-seq sequencing data reported in this study have been deposited in the ArrayExpress database under the accession number E-MTAB-16602.

Research involving human participants, their data, or biological material

Policy information about studies with human participants or human data. See also policy information about sex, gender (identity/presentation), and sexual orientation and race, ethnicity and racism.

Reporting on sex and gender	N/A
Reporting on race, ethnicity, or other socially relevant groupings	N/A
Population characteristics	N/A
Recruitment	N/A
Ethics oversight	N/A

Note that full information on the approval of the study protocol must also be provided in the manuscript.

Field-specific reporting

Please select the one below that is the best fit for your research. If you are not sure, read the appropriate sections before making your selection.

☒ Life sciences ☐ Behavioural & social sciences ☐ Ecological, evolutionary & environmental sciences

For a reference copy of the document with all sections, see nature.com/documents/nr-reporting-summary-flat.pdf

Life sciences study design

All studies must disclose on these points even when the disclosure is negative.

Sample size	No statistical methods were used to predetermine sample size for all experiments. Sample sizes were chosen based on previous studies in this field (Dai et al., Nature 2021; Ba et al., Nature 2020; Zhang et al., Nature 2024) that used similar sample sizes to generate reproducible results.
Data exclusions	No data was excluded from analysis.
Replication	All samples were analyzed with biological three repeats as detailed in the relevant text and figure legends. All attempts for replication were successful.
Randomization	Experiments were not randomized. Each experiment was performed with identified control and mutant strains. Randomization was not relevant to the study as the study does not involve participant groups.
Blinding	Investigators were not blinded to allocation during experiments and outcome assessment. Blinding was not possible as investigators need to verify the control and matched mutant strains before each experiment. Also, based on previous studies in this field, these assays do not require blinding.

Reporting for specific materials, systems and methods

We require information from authors about some types of materials, experimental systems and methods used in many studies. Here, indicate whether each material, system or method listed is relevant to your study. If you are not sure if a list item applies to your research, read the appropriate section before selecting a response.

Materials & experimental systems

n/a	Involved in the study
☐	☒ Antibodies
☐	☒ Eukaryotic cell lines
☒	☐ Palaeontology and archaeology
☐	☒ Animals and other organisms
☒	☐ Clinical data
☒	☐ Dual use research of concern
☒	☐ Plants

Methods

n/a	Involved in the study
☒	☐ ChIP-seq
☐	☒ Flow cytometry
☒	☐ MRI-based neuroimaging

Antibodies

Antibodies used

anti-B220-APC (eBioscience, #17-0452-83), 1:1000
anti-CD43-PE (BD Biosciences, #553271), 1:400
anti-IgM-FITC (eBioscience, #11-5790-81), 1:500
anti-B220-BV711 (BioLegend, Cat#103255), 1:300
anti-CD25-PE (BD PharmingenTM, Cat#561065), 1:300
anti-IgG1-FITC (BD Biosciences, Cat#553443), 1:500
anti-IgM-APC (Invitrogen, Cat#17-5790-82),1:500
anti-CD19-BV421 (BD Biosciences, Cat#562701), 1:300
anti-c-Kit-PE/Cy7 (eBioscienceTM, Cat#25-1171-81) , 1:300

Validation

anti-B220-APC (eBioscience, #17-0452-83), anti-CD43-PE (BD Biosciences, #553271) and anti-IgM-FITC (eBioscience, #11-5790-81) have been confirmed by FACS in published papers including (except this study): Dai, H.-Q. et al. Loop extrusion mediates physiological Igh locus contraction for RAG scanning. Nature 590, 338–343 (2021).

anti-B220-BV711 (BioLegend, Cat#103255), anti-CD25-PE (BD PharmingenTM, Cat#561065), anti-IgG1-FITC (BD Biosciences, Cat#553443), anti-IgM-APC (Invitrogen, Cat#17-5790-82), anti-CD19-BV421 (BD Biosciences, Cat#562701) and anti-c-Kit-PE/Cy7 (eBioscienceTM, Cat#25-1171-81) have been confirmed by FACS in published papers including(except this study): Hill, L. et al. Wapl repression by Pax5 promotes V gene recombination by Igh loop extrusion. Nature 584, 142-147 (2020).

Eukaryotic cell lines

Policy information about cell lines and Sex and Gender in Research

Cell line source(s)

The primary pre-B cells were derived from bone marrows of 4-8-week-old WT mice, Cer/Sis-deleted mice and iPSC-derived chimeras in both sex. The iPSC cell lines and derivatives were generated by reprogramming from splenic B cells, made in our lab. All immortalized v-Abl cell lines and derivatives were generated by retroviral infection of primary pro-B cells derived from RAG2-deficient; Em-Bcl2 transgenic male 129SV mice with pMSCV-v-Abl retrovirus, made in our lab. See Methods for details.

Authentication

All cell lines were authenticated by HTGTS-V(D)J-Seq using indicated baits, PCR genotyping and Sanger sequencing. See Methods for details. Sequences of pre-rearranged Vk/Jk segments and targeted genome modifications are listed in Supplementary Table 2. Sequences of all sgRNAs and oligos used are listed in Supplementary Table 3.

Mycoplasma contamination

All iPSC lines used for targeting and RAG-deficient blastocyst complementation injections were confirmed to be mycoplasma free. v-Abl cell lines were not tested for mycoplasma contamination.

Commonly misidentified lines
(See ICLAC register)

None

Animals and other research organisms

Policy information about studies involving animals; ARRIVE guidelines recommended for reporting animal research, and Sex and Gender in Research

Laboratory animals

We used 4-8-week-old WT mice and Cer/Sis-deleted 129SV mice, including both males and females, for isolating primary pre-B cells from bone marrow. We used 4-8-week-old iPSC-derived chimeras containing mixed genetic background of C57BL/6 and 129SV, including both males and females, for isolating primary immature B cells from bone marrow.

Wild animals

The study did not involve wild animals.

Reporting on sex

Both male and female mice were used in experiments.

Field-collected samples

The study did not involve samples collected from the field.

Ethics oversight

All mouse work were performed in compliance with all the relevant ethical regulations established by the Institutional Animal Care and Use Committee (IACUC) of Boston Children’s Hospital and under protocols approved by the IACUC of Boston Children’s Hospital.

Note that full information on the approval of the study protocol must also be provided in the manuscript.

Plants

Seed stocks	N/A
Novel plant genotypes	N/A
Authentication	N/A

Flow Cytometry

Plots

Confirm that:

☒ The axis labels state the marker and fluorochrome used (e.g. CD4-FITC).

☒ The axis scales are clearly visible. Include numbers along axes only for bottom left plot of group (a 'group' is an analysis of identical markers).

☒ All plots are contour plots with outliers or pseudocolor plots.

☒ A numerical value for number of cells or percentage (with statistics) is provided.

Methodology

Sample preparation	Single cell suspensions were derived from bone marrows of 4-8-week-old iPSC-derived chimeras, incubated in Red Blood Cell Lysing Buffer (Sigma-Aldrich, #R7757) to deplete the erythrocytes. Immature B cells were isolated by staining with anti-B220-BV711 (BioLegend, Cat#103255), anti-CD25-PE (BD PharmingenTM, Cat#561065), anti-IgG1-FITC (BD Biosciences, Cat#553443), anti-IgM-APC (Invitrogen, Cat#17-5790-82), anti-CD19-BV421 (BD Biosciences, Cat#562701) and anti-c-Kit-PE/Cy7 (eBioscienceTM, Cat#25-1171-81) antibodies for 30 minutes at 4 °C and then purified by FACS.
Instrument	BD FACSAria II
Software	FlowJo vX.0.7
Cell population abundance	The cell populations of Pre-B cells and Immature B cells in the BM marrow of iPSC-derived chimeras are indicated in Extended Data Fig.5 c, d.
Gating strategy	Lymphocyte population was gated by FACS side (SSC) and forward (FSC) scatters out of the total cells. Then B220+CD19+ B cells were gated and selected by FACS. IgG1+IgM- immature B cells were gated and shown in the left plot (Extended Data Fig.5 c, d). IgG1-IgM- B cells were also gated, and used for gating CD25+c-Kit- pre-B cells indicated in the right plot (Extended Data Fig.5 c,d).

☒ Tick this box to confirm that a figure exemplifying the gating strategy is provided in the Supplementary Information.
